# Temporal and spatial dose distribution of radiation pneumonitis after concurrent radiochemotherapy in stage III non-small cell cancer patients

**DOI:** 10.1186/s13014-017-0898-5

**Published:** 2017-11-02

**Authors:** Mohammed Alharbi, Stefan Janssen, Heiko Golpon, Michael Bremer, Christoph Henkenberens

**Affiliations:** 1 Department of Radio-Oncology, Medical School Hannover, Carl-Neuberg-Str. 1, 30625 Hannover, Germany; 2 Joint Practice Radiooncology Hannover, Rundestr. 10, 30161 Hannover, Germany; 3 Department of Pneumology, Medical School Hannover, Carl-Neuberg-Str. 1, 30625 Hannover, Germany; 4 Department of Radiation Oncology, University of Lübeck, Ratzeburger Ave. 160, 23562 Lübeck, Germany

**Keywords:** Symptomatic radiation pneumonitis, Chemoradiotherapy, Lung cancer, Temporal dose volume change

## Abstract

**Background and purpose:**

Radiation pneumonitis (RP) is the most common subacute side effect after concurrent chemoradiotherapy (CRT) for locally advanced non-small cell lung cancer. Several clinical and dose-volume (DV) parameters are associated with a distinct risk of symptomatic RP. The aim of this study was to assess the spatial dose distribution of the RP volume from first occurence to maximum volume expansion of RP.

**Material and methods:**

Between 2007 and 2015, 732 patients with lung cancer were treated in an institution. Thirty-three patients met the following inclusion criteria: an RP grade II after CRT and a radiation dose ≥60 Gy and no prior medical history of cardiopulmonary comorbidities. The images of the first chest computed tomography (CT) confirming the diagnosis of RP and the CT images showing the maximum expansion of RP were merged with the treatment plan. The RP volume was delineated within the treatment plan, and a DV analysis was performed to evaluate the lung dose volume areas in which the RP manifested over time and whether dose volume changes within the RP volume occurred.

**Results:**

A change from clinical diagnosis to maximum expansion of RP was observed as the RP at clinical appearance mainly manifested in the lower dose areas of the lung, whereas the RP volume at maximum expansion manifested in the higher dose areas, resulting in a significant shift of the assessed relative mean dose volume proportions within the RP volume. The mean relative dose volume proportion 0- ≤ 20 Gy decreased from 30.2% (range, 0–100) to 21.9% (range, 0–100; *p* = 0.04) at the expense of the dose volume > 40 Gy which increased from 39.2% (range, 0–100) to 49.8% (range, 0–100; *p* = 0.02), whereas the dose relative volume proportion > 20- ≤ 40 Gy showed no relevant change and slightly decreased from 30.6% (range, 0–85.7) to 28.3%, (range, 0–85.7; *p* = 0.34).

**Conclusion:**

We observed a considerable increase in the relative dose proportions within the RP volume from diagnosis to maximum volume extent from low dose zones below 20 Gy to zones above 40 Gy. Although the clinical impact on RP remains unknown, a reduction of healthy healthy lung tissue receiving >40 Gy (V40) might be an additional parameter for irradiation planning in lung cancer patients.

## Introduction

Concurrent chemoradiotherapy (CRT) is the standard treatment for locally advanced non-small cell lung cancer (NSCLC), and radiation pneumonitis (RP) is one of the major dose-limiting side effects. High radiation doses significantly improve local control but also increase the incidence of severe RP and lead to poor quality of life and, in a few cases, death [1–3]. Several heterogeneous reports have investigated the incidence of clinically significant RP grade ≥ 2 ranging from 10 to 30% after CRT with 60 to 70 Gray (Gy) and assessed numerous clinical and various dose volumetric (DV) parameters factors that are likely associated with clinically significant RP [4–9]. Since the introduction of three-dimensional (3D) treatment planning systems to the clinical routine, dose volume distributions of organs at risk can easily be assessed, and many studies have evaluated the association between DV parameters and the risk of RP [5, 7, 8]. Until now, no data assessing in which dose volume areas of the lung RP manifested at diagnosis and at maximum volume expansion have been available. Furthermore, it is unknown whether changes in relative dose volume proportions of the RP volume are related to volume changes.

Therefore, in the presented analysis, we assessed the spatial DV parameter changes in the RP volume at diagnosis and at maximum volume expansion over time.

## Material and methods

### Patient parameters

Between 2007 and 2015, 732 patients with lung cancer were treated in an institution and 33 of the 732 developed a RP grade II after CRT according to CTCAE v. 4.0 [10] and met the following inclusion criteria: no prior medical history of coronary heart disease requiring stent implantation or aortocoronary bypass surgery, known heart insufficiency, chronic obstructive pulmonary disease (COPD) or interstitial lung changes that are known to be associated with lung changes on lung imaging [11]. The patient and treatment characteristics are summarised in Table 1.

**Table 1:** Patient characteristics

	Patients (*n* = 33)
Median age (years, range)	68.0 (49–82)
Gender	
Male	21 (63.6%)
Female	12 (36.4%)
UICC-stage (TNM 7th ed.)	
IIIA	14 (42.4%)
IIIB	19 (57.6%)
Histological type	
Squamous cell carcinoma	16 (48.5%)
Adenocarcinoma	17 (51.5%)
Smoker status	
Non-smoker	8 (24.2%)
Smoker	28 (75.8%)
Chemotherapy	
Cisplatin/Vinorelbine	10 (30.3%)
Carboplatin/Paclitaxel	23 (69.7%)
Localisation	
Periphery	23 (69.7%)
Central	10 (30.3%)
Left	16 (48.5%)
Right	17 (51.5%)
Mean total lung dose (Gy)	15.7 (8.2–24.43)
Mean V20	25.1 (12.0–45.01)
Mean V30	17.3 (6.4–34.27)

### CRT

In all patients, radiotherapy was based on a planning CT (slice thickness: 3 mm) and three-3D treatment planning using Oncentra MasterPlan® (Nucletron/Elekta, Stockholm, Sweden). Gross tumour volume (GTV) was identified by computed tomography, PET scan, and bronchoscopy. Hilar and mediastinal lymph nodes with a short axis ≥ 1.0 cm or pretreatment PET scan with standardised uptake values (SUV) > 3 were included. The clinical target volume (CTV) was defined as GTV plus an individual margin up to 1.0 cm to account for microscopic tumour extension. For planning target volumes (PTVs), the CTVs were enlarged to allow for organ motion and setup variation and were expanded individually up to 1.0 cm in all directions. On-board cone-beam computed tomography was used at least weekly to confirm the correct patient positioning. Radiotherapy was carried out five times weekly at a daily single doses of 2.0 Gray (Gy). The radiation doses were 46–50.0 Gy to the mediastinum and 60–66 Gy to the primary tumour and pathological lymph nodes.

In general, irradiation was delivered using a conformal multifield technique with 6- to 10-MV photons of a linac accelerator. Quantitative dose-volume analyses were performed using cumulative dose-volume histograms (DVH) to keep the mean lung dose (MLD) below 20 Gy and mean V20 and V30 below 30 and 20%, respectively. The patients received either weekly concurrent carboplatin AUC2 plus paclitaxel 100 mg/m^2^ [12] or 2 cycles of cisplatin 20 mg/m^2^ day 1–4 plus oral vinorelbine 50 mg/m^2^ on days 1, 8, and 15 [13]. None of the patients underwent induction or consolidation chemotherapy.

### RP diagnosis, treatment and follow-up

The diagnosis of RP was based on the typical clinical symptoms such as new dyspnoea, non-productive cough, pleuritic chest pain and fever. A lung function test and a capillary blood gas test were also carried out. In all patients, computed tomography (CT) of the chest was performed within 3 days after presentation with RP symptoms to radiographically verify RP. CT scans of the chest were performed on a four-week basis until a substantial reduction in the pneumonitic ground glass opacities was observed. Thereafter, a CT scan of the chest and abdomen was routinely performed every 2 months. All patients underwent an oncological follow-up visit after the CT scan. Furthermore, the patients had bi-weekly clinical follow up visits until the steroids were tapered off completely (0.5 mg/kg bodyweight prednisolone with at least 40 mg initial dose per day). Every 5 days, the prednisolone dose was halved until the maintenance dose of 6 mg per day was attained, which was usually maintained for an additional 6 weeks.

### DVH analysis for temporal and spatial dose distribution

**Fig. 1:**
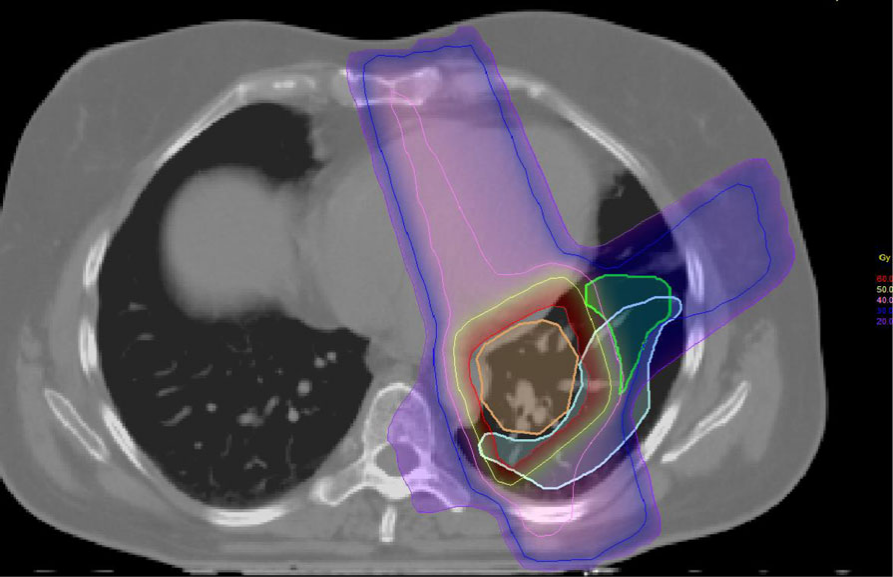
Example for the delineation of the RP volume. Two CT scans of the chest to confirm the clinical diagnosis and showing the maximum expansion of RP were fused with the treatment plan and both the pneumonitic lung areas at the time point of clinical appearance and at maximum expansion were delineated. In this example, the RP at the time point of clinical appearance (green contour) was located outside the 50 Gy isodose (yellow), mainly in lung areas with doses below 30 Gy. The larger RP volume at the time point of maximum expansion of RP (light blue contour) was also observed within the lung areas receiving more than 50 Gy (yellow 50 Gy isodose and red coloured the 60 Gy isodose) encompassing the bottom of the PTV (orange contour)

We reviewed all CT scans starting at the time point of first clinical occurrence of RP grade II until a significant reduction in pneumonitic areas was observed. We merged the images of the first chest CT that confirmed the diagnosis of RP after CRT and the CT images showing the maximum expansion of the typical pneumonitic ground glass opacities with the treatment plan using a planning treatment systems mutual algorithm. Am, JS, BM and CH delineated the pneumonitic areas using lung window of both CT scans at appearance and maximum expansion of RP lung changes (Fig. 1). In some cases the delineated volumes were modified on an individual basis to account for structural changes after CRT to avoid that former tumor areas after tumor shrinkage were incorrectly delineated as RP areas. A DVH analysis was then performed to assess in which dose areas of the lung the RP manifested at diagnosis and at maximum expansion to evaluate the relative dose volume proportions within the delineated RP volume at clinical appearance and at maximum expansion. The relative dose volume proportions of the patients were added to the relative mean dose volume proportions, which were used for statistical analysis.

### Statistical analysis

Statistical analysis was performed using a commercially available software package (SPSS V. 24.0 for Windows, IBM, NY, USA). A paired samples t-test for parametric parameters was carried out to assess DV changes at diagnosis and at maximum RP expansion. Furthermore, we investigated whether clinical parameters, abnormal lung function test and capillary blood gas parameters below the normal values at RP diagnosis had a statistically significant influence on the DV changes within the RP volume using a one-way repeated measures analysis of variance (rmANOVA).

## Results

**Fig. 2:**
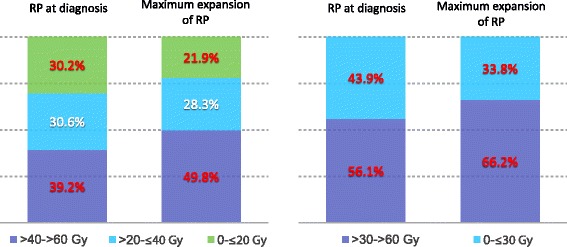
Bar charts showing an increase of the higher relative dose proportion of the RP volume from clinical appearance to maximum radiographic volume expansion after median 3.75 months (range, 2.0–7.75) after completion of radiotherapy. Statistically significant changes (red letters) for relative dose proportion less than 20 Gy and 40 Gy (left bar chart), as well as for doses less than 30 Gy towards doses above 30 (right bar chart), were observed. The dose span from 20 to 40 Gy showed an almost constant proportion of the pneumonitic lung volume of 30.6 and 28.3% at diagnosis and at maximum expansion, respectively (left bar chart)

RP manifested clinically at median of 1.5 (range, 0.5–4.5) months after CRT, and the maximum volume expansion of the ground glass opacities on the CT images were observed at a median 3.75 (range, 2.0–7.75) months after CRT. All patients responded well to corticosteroids and none of the patients developed increased symptoms (e.g. requiring oxygen) after therapy with corticosteroids was initiated. The RP volume at diagnosis was at a median of 80.8 ccm (range, 3.0–471.0) and increased significantly to 268.6 ccm (range, 19.9–674.0; p = < 0.001) at the time point of the greatest expansion after a median of 3.75 months after CRT. The mean dose within the pneumonitic lung areas was 33.4 Gy (range, 1.0–59.8) at diagnosis and was significantly higher with 37.8 Gy (range, 8.0–64.3; *p* = 0.026) at the time of maximum expansion. Furthermore, the mean peak dose within the pneumonitic areas was significantly increased from initial appearance (52.3 Gy; range, 1.6–64.3) to maximum expansion (57.7 Gy; range, 8.0–66.32; *p* = 0.015). We observed that the RP at diagnosis mainly manifested in the lower dose areas of the lung, whereas the main proportion of the RP at maximum expansion was located in the higher dose areas and led to shifts in the relative dose distribution of the RP volume. We observed a statistically significant decrease in the relative mean dose volume proportion 0- ≤ 20 Gy from 30.2% (range, 0–100) to 21.9 (range, 0–100; *p* = 0.04) and an increase >40 Gy from 39.2% (range, 0–100) to 49.8% (range, 0–100; *p* = 0.02), whereas the relative mean dose volume proportion of >20- ≤ 40 Gy showed a slight but not significant decrease from 30.6% (range, 0–85.7) to 28.3%, (range, 0–85.7; *p* = 0.34). Considering the relative mean dose volume proportions below and above 30 Gy, we also found a statistically significant increase in the relative dose volume proportions >30 Gy from 56.1% (range, 0–100) to 66.1% (range, 0–100; *p* = 0.03). The results of the statistical analysis are shown in Table 2 and graphically in Fig. 2.

**Table 2:** Proportions of the assessed relative dose proportions at diagnosis and at maximum radiographic expansion showing a statistically significant increase towards the higher dose areas >30 Gy and >40 Gy

Dose Interval (Gy)	Proportion (%, range) at Diagnosis of RP	Proportion (%, range) at maximum expansion of RP	*p*-Value
0- ≤ 20	30.2 (0–100)	21.9 (0–100)	0.04
>20- ≤ 40	30.6 (0–86)	28.3 (0–78)	0.34
>40	39.2 (0–100)	49.8 (0–95)	0.02
0- ≤ 30	43.9 (0–100)	33.8 (0–100)	0.03
>30	56.1 (0–100)	66.2 (0–100)	0.03

Regarding the results of the rmANOVA, none of the clinical parameters including side, localisation, sex, smoking status or chemotherapy regime had a statistically significant effect on the observed changes of the relative dose volume proportions of the RP volume. Patients with a vital capcity (VC) <100% had a statistically significantly greater increase in the relative proportions >30 Gy (*p* = 0.039) and >40 Gy (*p* = 0.044), and a significantly smaller relative proportion 0- ≤ 30 Gy (*p* = 0.043) compared to patients with a normal VC >100%. None of the other lung function parameters or capillary blood gas concentrations had a significantly effect on the observed changes of the relative dose volume proportions within the RP volume. Furthermore patients treated with a photon energy ≥10 megavoltage (MV) also had a significantly greater increase in the relative proportions >40 Gy (*p* = 0.033) compared to patients irradiated with 6 MV only. The results of the rmANOVA are presented in Table 3.

**Table 3:**
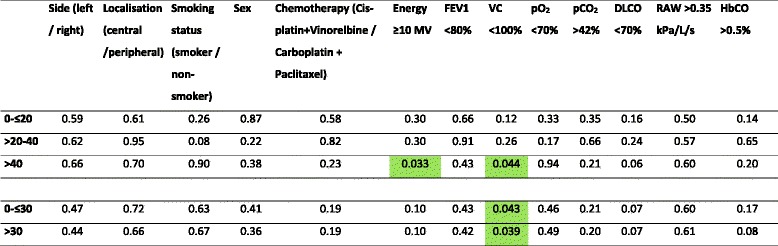
Results of the one-way repeated measures analysis of variance (rmANOVA) of the assessed clinical, lung function and blood gas parameters at diagnosis of RP parameters

Statistically significant results are highlighted with a green background

Abbreviation: *MV* Megavoltage, *FEV1* forced expiratory volume in 1 s; *VC* vital capacity, *pO*_*2*_ partial pressure of oxygen, *pCO*_*2*_ partial pressure of carbon dioxide, *DLCO* diffusion capacity of carbon monoxide, *RAW* airway resistance, *HbCO* fraction of carboxyhaemoglobin

## Discussion

RP is the most common severe and clinically relevant subacute side effect after radiotherapy in lung cancer with a reported incidence of symptomatic RP of approximately 10–30%. This range likely arises from different patient populations, a subjective RP scoring system and treatment-related factors such as radiotherapy technique and fractionation and chemotherapy [2, 7].

The application of oral steroids is recommended for treatment of symptomatic RP ≥ grade II. The therapy is not causal but merely symptomatic and does not influence the progression of RP to lung fibrosis. Several drugs, such as TNF-alpha or TGF-beta inhibitors, failed as causal treatments to interrupt inflammation progressing towards fibrosis, and none of these agents have been established as RP therapies [14]. Therefore, many studies have investigated patient- and treatment-related clinical parameters, such as comorbidities, concurrent chemotherapy and a variety of dose-volume parameters as risk factors for symptomatic RP [15–17]. All of these studies have suggested that certain clinical and dose volume parameters are associated with an increased risk or are even predictors of RP after radiotherapy. Thus far, to the best of our knowledge, no studies have reported changes in the dose areas of the lung when RP manifests with clinical symptoms or when RP had its greatest expansion. Therefore, there are no data on the associated changes of the relative dose volume proportions of the delineated RP areas from diagnosis to maximum expansion after definitive CRT for locally advanced NSCLC.

In the present analysis, we found that the delineated RP volume at clinical appearance was mainly located in lower dose areas of the lung, as the majority of the RP volume was formed by “low” mean dose volume proportions low dose volume, whereas the RP volume at maximum expansion was mainly located in the higher dose areas of the lung, resulting in shift for the mean relative dose proportions towards “high” dose volume proportions above 40 Gy. The mean relative proportion of the intermediate dose volume interval from 20 to 40 Gy remained almost unchanged, suggesting that the clinical course of RP, until the decline of the typical RP signs on chest CT scans, was mainly determined by the high dose areas of the lung in the treatment plan. This was also confirmed to a lesser extent for the dichotomous discrimination of mean relative dose volume proportions <30 and >30 Gy.

These findings emphasise that in addition to the typical DVH parameters, such as V20 < 30%, V30 < 20% and an MLD <22 Gy [17], which are associated with an increased incidence of symptomatic RP after combined-modality therapy for NSCLC, the volume of the lung receiving higher doses, in particular above 40 Gy (V40), should be taken into consideration and reduced as much as possible in irradiation planning. However, in some cases, the above-mentioned lung dose constraints have to be compromised to reach target volume coverage. Moreover, we do not know if smaller lung volumes receiving doses >40 Gy have either impact on the incidence of RP nor can we foreclose that this will reduce the RP volume, as this might be compensated by larger low dose-zones. Furthermore, the impact on the clinical symptoms is ambiguous, because the relative lower dose proportions 0- ≤ 20 Gy and >20- ≤ 40 Gy within the RP volume at clinical occurrence have been 30.2 and 30.6%, respectively. This might imply that the lung volumes >40 Gy may had no significant influence on symptoms at clinical appearance of RP.

Brierie et al. who showed that RP depends on sparing of total lung volumes at 40 Gy resulted in a significantly decreased incidence of RP 6 months after radiotherapy in a cohort of 579 NSCLC patients of which 449 (77.5%) had concurrent chemotherapy and a radiotherapy dose of at least 50 Gy [18]. Their results and our findings in the present analysis suggest that in patients with locally advanced NSCLC (UICC stage III) receiving CRT, the radiation oncologist should monitor the dose volume of the healthy lung tissue receiving 40 Gy and above because this is likely to have an impact on both the incidence and the volumetric expansion of symptomatic RP, and sparing the lung treatment with >40 Gy (V40) might be beneficial.

In addition to the above-mentioned classical dose constraints, several static and functional parameters are proposed to reduce the risk of RP.

Other factors include the V5 [19], the lung volume receiving a dose less than 5 Gy below 1500 ccm [16], the ipsilateral V20 and ipsilateral V30 [17], and functional differences in regional lung perfusion assessed by single photon emission CT lung perfusion scans [20], which are less common and were not used for irradiation planning for our patients. However, these parameters might have had an influence on the observed changes in the relative dose volume proportions of the RP volume from diagnosis to the maximum expansion RP. However, many these dosimetric variables tend to be very collinear, and therefore, differences among different dosimetric variables may be small leading to an unknown merit of decreasing one (e.g., lowering V20) at the expense of another (e.g., raising the V5) [21].

The limitations of our study include the inherent weaknesses of its retrospective nature. In addition, the relatively small patient sample size (*n* = 33) might have introduced statistical bias in this report. Nevertheless, the results seem to be robust due to the homogenous patient cohort, which included only medically fit patients with NSCLC treated with definitive CRT. Otherwise, no conclusion could be drawn for patients who had either mediastinal radiotherapy post lung surgery or sequential chemotherapy. The intention was to reduce possible confounders for the delineation of the ground-glass opacities of RP. Thus, we excluded patients with severe comorbidities, such as COPD or interstitial lung diseases, which may lead to an estimated misdiagnosis rate of up to 20% of RP [22]. This might be a reason why typical lung function parameters associated with symptomatic RP, such as decreased forced expiratory volume in 1 s (FEV1) and diffusion capacity for carbon monoxide (DLCO) [23, 24], showed no significant effect on the observed shift from low to high dose relative volume proportions, whereas our statistical analysis using rmANOVA supported the assumption that a vital capacity (VC) < 100% at diagnosis of RP might be an indicator for a relevant shift. Recently, Bradley et al. demonstrated that patients of the RTOG 0617 trial treated with intensity-modulated radiation therapy (IMRT) had symptomatic RP less often than patients treated with 3D–conformal CRT due to a significantly lower V20, and in general, IMRT allows for better protection of healthy lung tissue than 3D–RT [25]. Due to our results in which the majority of the RP volume at maximum expansion was located in lung areas with higher doses above 20 Gy, we also recommend IMRT for CRT in patients with NSCLC.

## Conclusion

We observed a considerable increase in the relative dose proportions within the RP volume from diagnosis to maximum volume extent from low dose zones below 20 Gy to zones above 40 Gy. The impact on incidence and severity of RP is still unknown. More clinical data are needed to assess the impact of these finding on the incidence and severity of RP. Nevertheless the volume of healthy lung tissue receiving >40 Gy (V40) might be an additional parameter for irradiation planning in lung cancer patients.
